# Implanted Penile Prosthetic Visualized During Focused Assessment with Sonography for Trauma Examination: A Case Report

**DOI:** 10.5811/cpcem.1407

**Published:** 2023-05-27

**Authors:** Kevin Chambers, Geoffrey Comp

**Affiliations:** * Creighton University School of Medicine, Department of Emergency Medicine, Phoenix, Arizona; † University of Arizona College of Medicine-Phoenix, Phoenix, Arizona

**Keywords:** case report, implanted penile prosthetic, point-of-care ultrasound, focused assessment with sonography for trauma (FAST)

## Abstract

**Introduction:**

This is a case report of an implanted penile prosthetic visualized during focused assessment with sonography for trauma (FAST) examination. The case represents a unique finding near the lateral bladder that may confound assessment of intraperitoneal fluid collections during initial assessment of trauma patients.

**Case Report:**

A 61-year-old Black male was brought to the emergency department from a nursing facility for evaluation after sustaining a ground-level fall. A FAST exam demonstrated an abnormal fluid collection anterior and lateral to the bladder, later identified as an implanted penile prosthetic.

**Conclusion:**

Focused assessment with sonography for trauma examinations are often performed on unidentified patients in a time-sensitive manner. Understanding of potential false-positive results is crucial to appropriate use of the tool. This report demonstrates a novel false-positive result that may be difficult to differentiate from a true intraperitoneal bleed.

## INTRODUCTION

The focused assessment with sonography for trauma (FAST) examination is a widely used point-of-care ultrasound tool to evaluate for intraperitoneal bleeding in trauma patients. It is frequently used as part of the initial assessment of traumatic injuries and may determine whether a patient has need for additional imaging such as computed tomography (CT) or surgical intervention such as an exploratory laparotomy.^1^ The sensitivity and specificity of the FAST exam have been widely studied and are reportedly as high as 96% and 98%, respectively.^2^,^3^ The FAST exam uses a series of standardized sonographic windows that identify regions where intraperitoneal fluid tends to accumulate due to being dependent areas. The lateral aspects of the bladder are one region where such fluids can be visualized. Numerous false-positive FAST results have been described in the literature including ascites, gastric contents, and perinephric fat pads.^4^ In this case report, we describe a potential false-positive FAST exam due to an implanted penile prosthetic in a patient presenting after a fall.

## CASE REPORT

A 61-year-old Black male was brought to the emergency department from a nursing facility for evaluation after sustaining a ground-level fall. The patient, who ambulated with a walker, had a past medical history of hypertension, diabetes, mild dementia, and cerebrovascular accident (CVA) with residual right-sided spastic hemiplegia. He had a witnessed fall while attempting to transfer from the bed to his walker, falling forward into the walker and onto the ground. No loss of consciousness or use of anticoagulation were reported.

Upon arrival, the patient was noted to be alert and oriented to person, place, and time; however, he did have some challenge relaying recent historical details. The patient’s initial vital signs were notable for temperature of 36.4° Celsius, heart rate of 109 beats per minute, blood pressure of 124/78 millimeters of mercury, respiratory rate of 18 breaths per minute, and oxygen saturation of 96% on room air. He was protecting his airway, with clear and equal breath sounds bilaterally. Heart sounds were non-muffled, and his abdomen was soft. Pelvis was stable to compression, and the patient had spastic motor function of all extremities consistent with his known CVA.

**Image 1:**
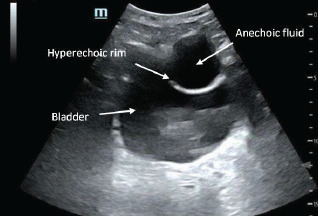
Point-of -care ultrasound image of anechoic fluid collection with hyperechoic rim lateral to bladder in transverse view.

Secondary survey revealed a superficial laceration to the right forehead and scattered abrasions over the anterior chest and abdomen. Due to evidence of abdominal trauma, a FAST exam was performed that revealed no free fluid in the right upper quadrant, left upper quadrant, or in subxiphoid views. However, the suprapubic view demonstrated an abnormal anechoic fluid collection anterior and lateral to the bladder (Image 1). The collection was visualized in transverse and sagittal views.

**Image 2:**
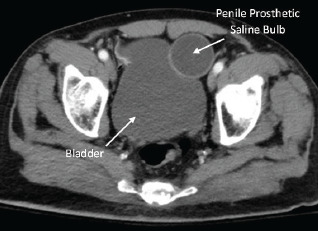
Computed tomography image of penile prosthetic saline bulb reservoir in transverse pelvic view.

Vital signs remained stable, and the patient’s abdomen remained soft and non-tender. He was not able to provide further clarification of the abnormal finding during initial questioning secondary to presumed dementia. During further evaluation of his abnormal ultrasound findings, genitourinary surgical scars were noted to the groin and scrotum. Upon chart review, we found a CT of the abdomen and pelvis with intravenous contrast that had previously identified the findings as a prosthetic penile implant (Image 2). Ultimately, the patient was cleared by trauma service following negative imaging of the head. No further evaluation of potential intraperitoneal bleeding was deemed necessary. The laceration to his forehead was repaired at bedside, and the patient was transported back to the nursing facility at his neurologic baseline.

*CPC-EM Capsule*

What do we already know about this clinical entity?

*Focused Assessment with Sonography for Trauma (FAST) examinations utilize a series of standardized sonographic windows that identify regions where intraperitoneal fluid tends to accumulate in dependent areas.*

What makes this presentation of disease reportable?

*This report demonstrates novel false positive images that may be difficult to differentiate from a true intraperitoneal bleed.*

What is the major learning point?

*Awareness of implanted penile prosthetic design and function is important to avoid false positive FAST examinations.*

How might this improve emergency medicine practice?

*FAST examinations are performed in a time sensitive manner and understanding of potential false positive results is crucial to appropriate use of the tool.*

## DISCUSSION

**Image 3:**
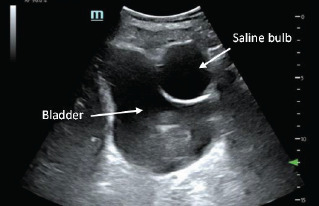
Point-of-care ultrasound image of saline reservoir in pelvic sagittal view.

In this presented case, the hyperechoic lucency with anechoic center is a reservoir for an implanted penile prosthetic (Image 3). Penile prosthetics are performed for a variety of reasons; they most commonly occur following prostatic surgical procedures to maintain sexual function and often incorporate discrete surgical scars for cosmetic purposes.^5^ There are approximately 20,000 such devices implanted annually.^6^ While many types of devices exist, this particular device encompassed cylindrical shafts placed within the corpus cavernosum, a pump mechanism, and a separate saline balloon reservoir implanted surgically in the inferior abdomen beneath the transversalis fascia.

The saline balloon can appear similar to a Foley catheter balloon but will be located outside the bladder wall. The reservoir is filled with saline and can change shape via the pump diverting pressure to the cylinder system.^7^ Therefore, the reservoir may not always appear as well circumscribed and could confound the FAST exam or even be interpreted as a false positive. Awareness of the implant design and function is important to avoid false-positive FAST exams. Typically, intraperitoneal fluid collects in the most dependent portions of the abdomen making isolated findings near the lateral bladder unusual.

## CONCLUSION

The case represents a unique finding near the lateral bladder that may confound assessment of intraperitoneal fluid collections. Focused assessment with sonography exams are often performed on unidentified patients in a time-sensitive manner; an understanding of potential false-positive results is crucial to appropriate use of the tool. Obtaining additional history from available sources and performing focused physical assessments can help explain potential false-positive FAST exams and avoid unnecessary interventions. Imaging tools alone should never replace a thorough history and physical exam.

The case we report here occurred in a patient who was hemodynamically stable with an overall low suspicion for intraperitoneal injury. Therefore, additional chart review allowed for identification of the abnormal ultrasonographic finding. However, in the unstable patient this finding may confound the diagnosis or resuscitation attempts or lead to potentially dangerous invasive measures.
